# Interobserver variability of ultrasound measurements for the differential diagnosis of uterine prolapse and cervical elongation without uterine prolapse

**DOI:** 10.1007/s00192-021-04980-y

**Published:** 2021-10-07

**Authors:** José Antonio García-Mejido, Zenaida Ramos Vega, Alberto Armijo Sánchez, Ana Fernández-Palacín, Carlota Borrero Fernández, José Antonio Sainz Bueno

**Affiliations:** 1 Department of Obstetrics and Gynecology, Valme University Hospital, Seville, Spain; 2 Department of Obstetrics and Gynecology, University of Seville, Seville, Spain; 3 Department of Obstetrics and Gynecology, Nuestra Señora de la Merced Hospital, Seville, Spain; 4 Biostatistics Unit, Department of Preventive Medicine and Public Health, University of Seville, Seville, Spain

**Keywords:** 3D transperineal ultrasound, Pelvic organ prolapse, Uterine prolapse, Cervical elongation

## Abstract

**Objectives:**

Our study aims to determine the interobserver variability of different ultrasound measurements (pubis-cervix distance, pubis-uterine fundus distance, and pubis-Douglascul-de-sac distance) previously analyzed for the ultrasound differential diagnosis of uterine prolapse (UP) and cervical elongation CE without UP.

**Materials and methods:**

We conducted a prospective observational study with 40 patients scheduled to undergo surgical correction of UP and CE without UP. All patients underwent pelvic floor ultrasound examination by an examiner (E1) who acquired ultrasound images. Using these images, E1 measured the distances for the ultrasound differential diagnosis of UP and CE without UP, and these distances were compared with those measured by the other examiner (E2). Values were analyzed by calculating ICCs with 95% CIs.

**Results:**

For UP, excellent reliability was obtained for all measurements except the pubis-Douglascul-de-sac measurement at rest, which was moderate (ICC 0.596;* p* = 0.028) and for the difference between the pubis-Douglascul-de-sac measurement at rest and during the Valsalva maneuver, which was good (ICC 0.691;* p* < 0.0005). For CE without UP, interobserver reliability was excellent for all measurements analyzed except the pubis-cervix measurement during the Valsalva maneuver, which was moderate (ICC 0.535;* p* = 0.052) and for the pubis-Douglascul-de-sac measurement at rest, which was good (ICC 0.768;* p* < 0.0005).

**Conclusions:**

There is excellent interobserver reliability in measurements of the difference in the distance from the pubic symphysis to the uterine fundus at rest and during the Valsalva maneuver for both UP and CE without UP, which are used for the ultrasound differential diagnosis of UP and CE without UP.

## Introduction

Pelvic organ prolapse (POP) is a common condition whose surgical repair was the most commonly performed hospital procedure in women over 70 years of age from 1979 to 2006 [1]. However, POP corrective surgery has been linked to a recurrence rate of up to 30% after initial surgery [2, 3], and reintervention is needed in up to 50% of patients who have undergone at least two previous surgical procedures to repair prolapse [4]. Therefore, accurate presurgical assessment is crucial to improve surgical outcomes.

Transperineal ultrasound has been established as a useful complementary test to evaluate POP. Different cutoff points have been defined for the posteroinferior aspect of the pubic symphysis below which POP is significant, that is, ≥ 10 mm for the anterior compartment and ≥ 15 mm for the middle and posterior compartments [5, 6]. Furthermore, ultrasound has been used for the differential diagnosis of the different conditions found in the anterior and posterior compartments [7–10]. However, although ultrasound differential diagnosis of the anterior and posterior compartment POP is well defined [7–10], only one study has been found that describes the ultrasound differential diagnosis of uterine prolapse (UP) and cervical elongation (CE) without UP [11] for the middle compartment. The clinical difference between UP and CE without UP is that DeLancey level I (the cardinal-uterosacral ligament complex) is relatively intact in CE without UP. Therefore, the differentially diagnosed could be made using the pelvic organ prolapse quantification system (POP-Q) [12], because it assesses the position of the DeLancey level I in the POP. The study conducted by García et al. [11] to differentiate UP and CE without UP described different measurements, such as the distance from the pubis to the cervix, the uterine fundus, and the Douglas cul-de-sac, along with the difference in these measurements when taken at rest and during the Valsalva maneuver, for the differential diagnosis of UP and CE without UP [11]. They concluded that a difference of ≥ 15 mm in the distance from the pubis to the uterine fundus at rest and during the Valsalva maneuver is useful to differentiate UP from CE without UP by ultrasound [11]. However, the study did not refer to the interobserver reproducibility of these measurements, which is a requirement if it is to become a clinically useful test. Therefore, our study aims to determine the interobserver variability of the different ultrasound measurements (pubis-cervix distance, pubis-uterine fundus distance, and pubis-Douglascul-de-sac distance) previously analyzed for the ultrasound differential diagnosis of UP and CE without UP.

## Materials and methods

Prospective observational study with 40 (20 had UP and 20 had CE without UP) consecutively recruited patients scheduled to undergo surgical correction of UP and CE without UP June 1, 2018, and November 31, 2019.

All patients were clinically evaluated by a surgeon specializing in pelvic floor pathology who conducted a standardized clinical examination using the International Continence Society Pelvic Organ Prolapse Quantification (ICS POP-Q) system to assess pelvic organ prolapse [13]. UP was defined as stage 2 or greater apical compartment prolapse, and CE without UP was defined as C point ≥ 0, a D point ≤ − 4 and an estimated cervical length ≥ 5 cm on pelvic examination.

Image volumes were acquired by an expert (E1) in pelvic floor ultrasound with more than 5 years of experience in pelvic floor ultrasound studies who was blinded to the results of the clinical examination. The 3/4D ultrasound images were acquired from the mean sagittal plane images, as described above [14], using a Toshiba Aplio 500 ultrasound (Tokyo, Japan) with a convex 6–8-MHz volumetric probe. Two volume measurements were taken for each patient: at rest and with the Valsalva maneuver (held for a minimum of 6 s [14]). Offline analyses of the ultrasound volumes were then performed.

The analysis of the ultrasound volumes was conducted by E1 and a different examiner (E2) who had expertise in capturing and processing 3/4D images of the pelvic floor. Before starting the image analysis, E2 was provided with audiovisual and written materials specifying how to perform the appropriate measurements (pubis-cervix distance, pubis-uterine fundus distance, and pubis-Douglascul-de-sac distance) previously analyzed for the ultrasound differential diagnosis of UP and CE without UP [11]. The previously established measurement criteria were followed to ensure a stable reference line [15]. The pelvic organ descent was measured relative to the posteroinferior margin of the pubic symphysis [6] in the midsagittal plane in reference to the uterine fundus (defined as the hyperechogenic line most distal to the pubis from the uterine fundus), the Douglas cul-de-sac (defined by the hyperechogenic line of the peritoneal fold at the uterine insertion) and the cervix (defined by the most descended hyperechogenic point of the uterine cervix) at rest and during the Valsalva maneuver [15]. Measurements above the posteroinferior margin of the pubic symphysis were defined as negative values, and measurements below it were defined as positive values (11) [15].

### Statistical analysis

**Fig. 1:**
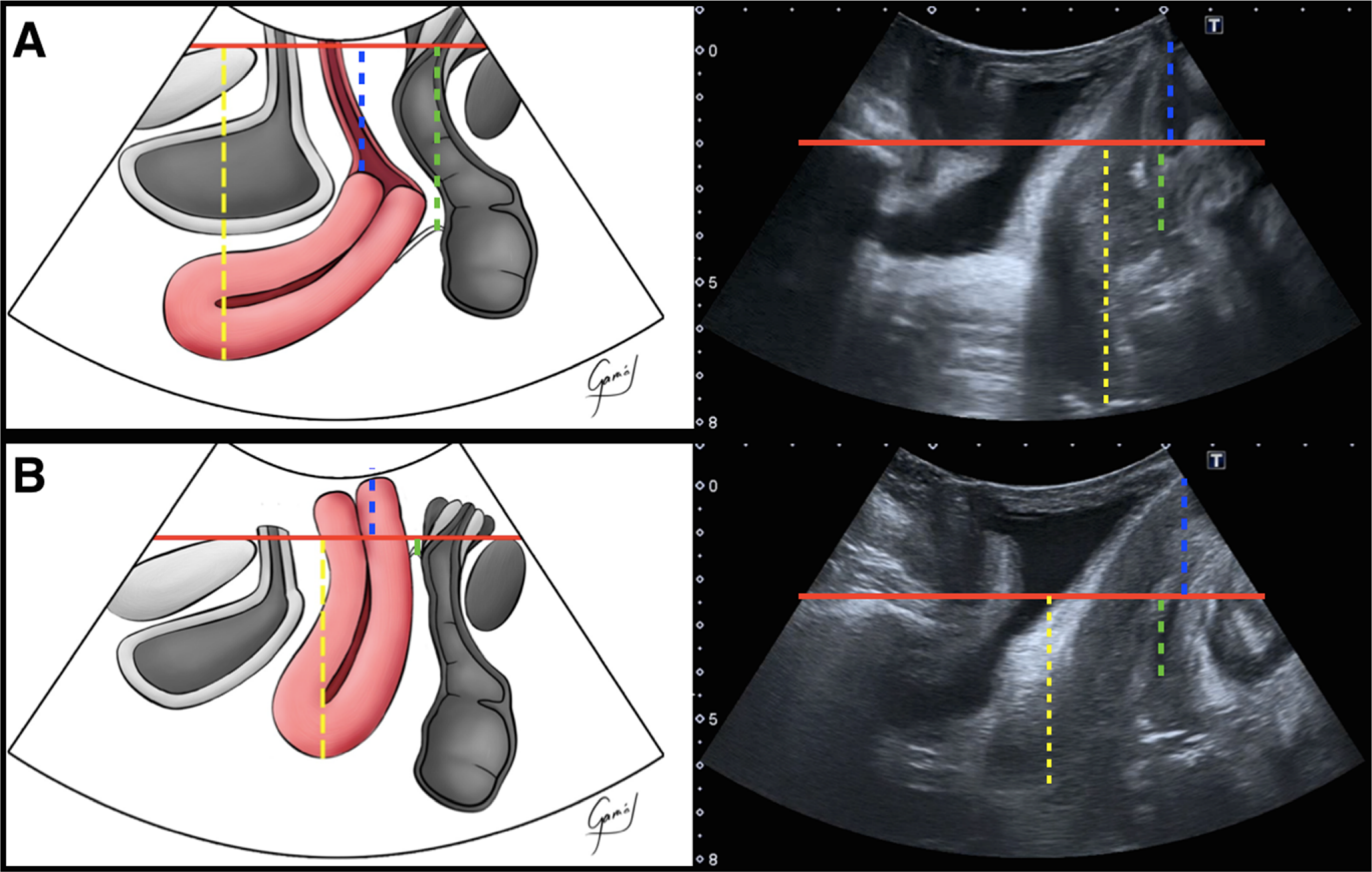
Midsagittal plane at rest (**A**) and during the Valsalva maneuver (**B**).* Red line* posteroinferior margin of the pubis;* blue line*pubis–cervix distance;* yellow line*pubis–uterine fundus distance;* green line*pubis–pouch of Douglas distance

The sample size was determined to estimate the intraclass correlation coefficient (ICC) as a measure of the reliability of measurements of the same subjects made using different methods. To calculate the sample size, we assumed an expected ICC value of 0.60 in the worst-case scenario (based on previous experience), a 95% confidence level, an accuracy or amplitude range of 0.2, and two repetitions of the measurements/observer. To meet these requirements, we needed to include at least 40 women. The values were analyzed by calculating ICCs with 95% CIs; an ICC value of < 0.2 was considered poor, 0.21–0.40 was considered fair, 0.41–0.60 was considered moderate, 0.61–0.80 was considered good, and 0.81–1.00 was considered excellent reliability [16]. The Bland–Altman 95% limits of agreement (LOA) method [16] was used to assess the mean difference between observers (“bias”). To test for significant bias, the 95% CI for the bias in each case was used to determine whether the bias differed from zero. Statistical analysis was performed using IBM SPSS Statistics 26 software Fig. 1.

### Ethical approval

The study (1259-N − 20) was approved by the local ethics and research committees.

## Results

Of the 40 patients included, 20 had UP, and the other 20 had CE without UP. Table 1 presents a comparison of the two examiners’ measurements. In the UP group, statistical differences between the two examiners were found only for the pubis-cervix distance during the Valsalva maneuver (20.6 ± 11.1 vs. 17.7 ± 8.1;* p* = 0.011), the pubis-uterine fundus distance at rest (− 66.5 ± 10.1 vs. − 65.5 ± 10.0;* p* = 0.023), the pubis-Douglascul-de-sac distance at rest (− 33.7 ± 17.7 vs. − 38.2 ± 10.1;* p* = 0.044) and during the Valsalva maneuver (− 14.5 ± 12.3 vs. − 17.3 ± 13.8;* p* = 0.045) and for the difference in the pubis-uterine fundus distance between the rest and Valsalva conditions (− 24.2 ± 12.1 vs. − 23.2 ± 11.9;* p* = 0.023). In the CE without UP group, statistical differences between the two examiners were found for the pubis-Douglascul-de-sac distance during the Valsalva maneuver (− 26.9 ± 24.2 vs. − 34.6 ± 25.3;* p* = 0.035).

**Table 1:** Comparison of the different measurements between the two examiners according to uterine prolapse or cervical elongation without uterine prolapse

	Uterine prolapse		*p* value	95% CI	Cervical elongation without uterine prolapse		*p* value	95% CI
	Mean (SD)				Mean (SD)			
	E1	E2			E1	E2		
Pubis-cervix measurement								
Rest	3.8 ± 11.6	3.0 ± 10.9	0.261	− 0.59; 2.06	7.6 ± 10.1	8.5 ± 9.7	0.289	− 2.69; 0.85
Valsalva	20.6 ± 11.1	17.7 ± 8.1	0.011	0.76; 5.12	16.3 ± 5.7	17.1 ± 8.7	0.815	− 4.32; 3.44
Pubis-uterine fundus measurement								
Rest	− 66.5 ± 10.1	− 65.5 ± 10.0	0.023	− 1.88; − 1.59	− 70.9 ± 17.1	− 70.1 ± 16.5	0.111	− 1.84; 0.21
Valsalva	− 42.3 ± 14.3	− 42.3 ± 13.7	0.933	− 1.15; 1.06	− 62.2 ± 30.8	− 62.3 ± 30.5	0.970	− 1.48; 1.53
Pubis-Douglas cul-de-sac measurement								
Rest	− 33.7 ± 17.7	− 38.2 ± 10.1	0.044	− 5.00; − 0.10	− 39.7 ± 12.0	− 43.1 ± 9.9	0.122	− 1.01; 7.93
Valsalva	− 14.5 ± 12.3	− 17.3 ± 13.8	0.045	− 4.50; − 0.05	− 26.9 ± 24.2	− 34.6 ± 25.3	0.035	− 12.50; − 0.2
Pubis-cervix measurement. Difference between rest and Valsalva	− 16.9 ± 15.4	− 14.7 ± 12.5	0.145	− 5.26; 0.84	− 9.1 ± 9.6	− 8.6 ± 8.6	0.764	− 3.82; 2.85
Pubis-uterine fundus measurement. Difference between rest and Valsalva	− 24.2 ± 12.1	− 23.2 ± 11.9	0.023	− 1.88; − 1.59	− 8.7 ± 23.8	− 7.9 ± 24.2	0.111	− 1.84; 0.21
Pubis-Douglas cul-de-sac measurement. Difference between rest and Valsalva	− 19.2 ± 19.2	− 20.9 ± 16.2	0.665	− 6.38; 9.78	− 12.7 ± 28.2	− 8.5 ± 27.6	0.251	− 11.67; 3.24

Table 2 shows the interobserver results for the different measurements of E1 and E2 for the 40 cases studied. For UP, excellent reliability was obtained for all measurements except the pubis-Douglascul-de-sac distance at rest, which showed moderate reliability (ICC 0.596; *p* = 0.028), and for the difference in the pubis-Douglascul-de-sac distance in the rest and Valsalva conditions, which showed good reliability (ICC 0.691; *p* < 0.0005). For CE without UP, interobserver reliability was excellent for all of the measurements analyzed except the pubis-cervix distance during the Valsalva maneuver, which showed moderate reliability (ICC 0.535;* p* = 0.052), and the pubis-Douglascul-de-sac distance at rest, which showed good reliability (ICC 0.768;* p* < 0.0005).

**Table 2:** Interobserver variability between the two examiners according to uterine prolapse or cervical elongation without uterine prolapse

	Uterine prolapse		*p* value	Cervical elongation without uterine prolapse		*p* value
	ICC	95% CI		ICC	95% CI	
Pubis-cervix measurement						
Rest	0.984	0.960–0.994	< 0.0005	0.962	0.904–0.985	< 0.0005
Valsalva	0.939	0.845–0.976	< 0.0005	0.535	− 0.174–0.816	0.052
Pubis-uterine fundus measurement						
Rest	0.992	0.979–0.997	< 0.0005	0.996	0.089–0.998	< 0.0005
Valsalva	0.993	0.982–0.997	< 0.0005	0.997	0.993–1.00	< 0.0005
Pubis-Douglas cul-de-sac measurement						
Rest	0.596	− 0.02–0.840	0.028	0.768	0.415–0.908	< 0.0005
Valsalva	0.939	0.846–0.976	< 0.0005	0.876	0.687–0.951	< 0.0005
Pubis-cervix measurement. Difference between rest and Valsalva	0.943	0.856–0.977	< 0.0005	0.820	0.545–0.929	< 0.0005
Pubis-uterine fundus measurement. Difference between rest and Valsalva	0.994	0.985–0.998	< 0.0005	0.998	0.995–0.999	< 0.0005
Pubis-Douglas cul-de-sac measurement. Difference between rest and Valsalva	0.691	0.220–0.878	< 0.0005	0.911	0.775–0.965	< 0.0005

## Discussion

This is the first study to describe the interobserver variability for ultrasound measurements (pubis-cervix distance, pubis-uterine fundus distance, and pubis-Douglascul-de-sac distance) previously analyzed for the differential diagnosis of UP and CE without UP [11]. Excellent reliability was found for the difference between the pubis-uterine fundus distance at rest and during the Valsalva maneuver for both UP (ICC 0.994;* p* < 0.0005) and CE without UP (ICC 0.998;* p* < 0.0005). In fact, the difference in the pubis-uterine fundus distance was the best parameter for the ultrasound differential diagnosis of UP and CE without UP. It has been described that a ≥ 15 mm difference in the pubis-uterine fundus distance at rest and during the Valsalva maneuver is useful for differentiating UP from CE without UP by ultrasound (sensitivity 75%; specificity 95%; positive predictive value 86%; negative predictive value 89%) [11].

Excellent interobserver variability has been previously described for 3–4D pelvic floor ultrasound measurements of the levator hiatus area [17]. These data are consistent with those described by van Veelen for the same measurements, with interobserver variability ranging from good to excellent between the first and second training sessions (ICCs 0.62–0.83 and 0.71–0.89, respectively, for the anteroposterior diameter, transverse diameter, and levator hiatus area at rest, during contraction and during the Valsalva maneuver) [18]. Other authors have also reported excellent reliability for the dimensions of the anteroposterior diameter, transverse diameter, and levator hiatus area at rest and during contraction [19–23]. However, excellent-to-moderate reliability has been established for the anteroposterior diameter and levator hiatus area during the Valsalva maneuver [20–23].

Encouraging results have also been reported for 2D pelvic floor ultrasound, with good interobserver correlations for different parameters, indicating that multicompartment pelvic floor ultrasound is a reliable tool for the anatomical assessment of pelvic floor measurements and POP [24]. Interobserver variability of 0.87 (95% CI 0.82–0.90) for the diagnosis of middle compartment prolapse has been specified [24]. These data are consistent with those previously described after 4–5 days of training, which indicated excellent reliability for the measurement of bladder neck descent (ICC 0.81) and cystocele descent (ICC 0.89) as well as good reliability for the assessment of uterine descent (ICC 0.74), rectal descent (ICC 0.76) and rectocele depth (ICC 0.75) [25] analyzed with 2 D ultrasound. Our data are consistent with those previously described in the literature, showing high agreement for the difference in the pubis-uterine fundus distance at rest and during the Valsalva maneuver in the differential diagnosis of UP and CE without UP [11].

The main strength of our study is that it is the first to describe interobserver variability in ultrasound measurements that is useful for the ultrasound differential diagnosis of UP and CE without UP [11]. Furthermore, we have observed a better interobserver variability for the diagnosis of uterine prolapse than that previously described with the clinical examination. This would help to ensure greater safety during the presurgical diagnosis of this pathology [25]. However, our main limitation is that we did not assess the learning process of E2, unlike previous studies that described the learning time required by evaluators [26]. Additionally, all of the ultrasound images used for the analysis were captured by E1, which may justify the results obtained. In future studies, it could be interesting to analyze interobserver variability in the acquisition and measurement of the different parameters that we analyzed.

In conclusion, there is excellent interobserver reliability for the measurement of the difference in the pubis-uterine fundus distance between rest and Valsalva conditions in both UP and CE without UP, supporting the ultrasound differential diagnosis of UP and CE without UP.

## Abbreviations

POP: pelvic organ prolapse
UP: uterine prolapse
CE: cervical elongation
POP-Q: pelvic organ prolapse quantification system
ICS POP-Q: International Continence Society Pelvic Organ Prolapse Quantification
E1: expert
E2: different examiner
ICC: intraclass correlation coefficient

## References

[1] Oliphant SS, Jones KA, Wang L, Bunker CH, Lowder JL (2010). Trends over time with commonly performed obstetric and gynecologic inpatient procedures. Obstet Gynecol.

[2] Olsen AL, Smith VJ, Bergstrom JO, Colling JC, Clark AL (1997). Epidemiology of surgically managed pelvic organ prolapse and urinary incontinence. Obstet Gynecol.

[3] Lavelle RS, Christie AL, Alhalabi F, Zimmern PE (2016). Risk of prolapse recurrence after native tissue anterior vaginal suspension procedure with intermediate to long-term follow-up. J Urol.

[4] Whiteside JL, Weber AM, Meyn LA, Walters MD (2004). Risk factors for prolapse recurrence after vaginal repair. Am J Obstet Gynecol.

[5] Dietz HP, Lekskulchai O (2007). Ultrasound assessment of prolapse: the relationship between prolapse severity and symptoms. Ultrasound Obstet Gynecol.

[6] Shek KL, Dietz HP (2015). What is abnormal uterine descent on translabial ultrasound?. Int Urogynecol J.

[7] Eisenberg VH, Chantarasorn V, Shek KL, Dietz HP (2010). Does levator ani injury affect cystocele type?. Ultrasound Obstet Gynecol.

[8] Green TH (1975). Urinary stress incontinence: differential diagnosis, pathophysiology, and management. Am J Obstet Gynecol.

[9] Chantarasorn V, Dietz HP (2012). Diagnosis of cystocele type by clinical examination and pelvic floor ultrasound. Ultrasound Obstet Gynecol.

[10] Dietz HP, Steensma AB (2005). Posterior compartment prolapse on two-dimensional and three-dimensional pelvic floor ultrasound: the distinction between true rectocele, perineal hypermotility and enterocele. Ultrasound Obstet Gynecol.

[11] García-Mejido JA, Ramos-Vega Z, Armijo-Sánchez A, Fernández-Palacín A, García-Jimenez R, Sainz JA. Differential diagnosis of middle compartment pelvic organ prolapse with transperineal ultrasound. Int Urogynecol J. 2021;23. 10.1007/s00192-020-04646-1. 10.1007/s00192-020-04646-1 33484288

[12] Bump RC, Mattiasson A, Bø K, Brubaker LP, DeLancey JO, Klarskov P (1996). The standardization of terminology of female pelvic organ prolapse and pelvic floor dysfunction. Am J Obstet Gynecol.

[13] Abrams P, Cardozo L, Fall M, Griffiths D, Rosier P, Ulmsten U (2002). The standardisation of terminology of lower urinary tract function: report from the standardisation sub-committee of the International Continence Society. Neurourol Urodyn.

[14] García-Mejido JA, Bonomi-Barby MJ, Armijo-Sanchez A, Borrero-Fernández C, Castro-Portillo L, Vargas-Broquetas M, Sainz JA. Methodology for the transperineal ultrasound imaging of the pelvic floor. Clin Invest Ginecol Obstet. 2020. 10.1016/j.gine.2020.09.008.

[15] Dietz H (2004). Ultrasound imaging of the pelvic floor. Part 1: two-dimensional aspects. Ultrasound Obstet Gynecol.

[16] Bland JM, Altman DG (1986). Statistical methods for assessing agreement between two methods of clinical measurement. Lancet.

[17] García-Mejido JA, Fernández-Palacín A, Bonomi-Barby MJ, De la Fuente VP, Iglesias E, Sainz JA (2020). Online learning for 3D/4D transperineal ultrasound of the pelvic floor. J Matern Fetal Neonatal Med.

[18] Van Veelen GA, Schweitzer KJ, Van der Vaart CH (2013). Reliability of pelvic floor measurements on three- and four-dimensional ultrasound during and after first pregnancy: implications for training. Ultrasound Obstet Gynecol.

[19] Braekken IH, Majida M, Engh ME (2009). Test–retest reliability of pelvic floor muscle contraction measured by 4D ultrasound. Neurourol Urodyn.

[20] Majida M, Braekken IH, Umek W (2009). Interobserver repeatability of three- and four-dimensional transperineal ultrasound assessment of pelvic floor muscle anatomy and function. Ultrasound Obstet Gynecol.

[21] Siafarikas F, Staer-Jensen J, Braekken IH (2013). Learning process for performing and analyzing 3D/4D 615 transperineal ultrasound imaging and interobserver reliability study. Ultrasound Obstet Gynecol..

[22] Braekken IH, Majida M, Ellstrøm-Engh M (2008). Test–retest and intra-observer repeatability of two-, three- and four-dimensional perineal ultrasound of pelvic floor muscle anatomy and function. Int Urogynecol J.

[23] Dietz HP, Shek C, Clarke B (2005). Biometry of the pubovisceral muscle and levator hiatus by three-dimensional pelvic floor ultrasound. Ultrasound Obstet Gynecol.

[24] Lone F, Sultan AH, Stankiewicz A, Thakar R (2016). Interobserver agreement of multicompartment ultrasound in the assessment of pelvic floor anatomy. Br J Radiol.

[25] Kobak WH, Rosenberger K, Walters MD (1996). Interobserver variation in the assessment of pelvic organ prolapse. Int Urogynecol J.

[26] Dietz HP, Rojas RG, Shek KL (2014). Postprocessing of pelvic floor ultrasound data: how repeatable is it?. Aust N Z J Obstet Gynaecol.

